# Supplementary material for: Direct Identification of Urinary Tract Pathogens by MALDI-TOF/TOF Analysis and De Novo Peptide Sequencing
Source: Molecules. 2022 Aug 25;27(17):5461. doi: 10.3390/molecules27175461 (PMC9457756; doi:10.3390/molecules27175461)
Supplement: Supplementary file 1 [file molecules-27-05461-s001.zip › molecules-1869626-supplementary.pdf]

SAMPLE PREPARATION (Step 1)

Isolation of proteins from the urine sample and FASP trypsin digestion

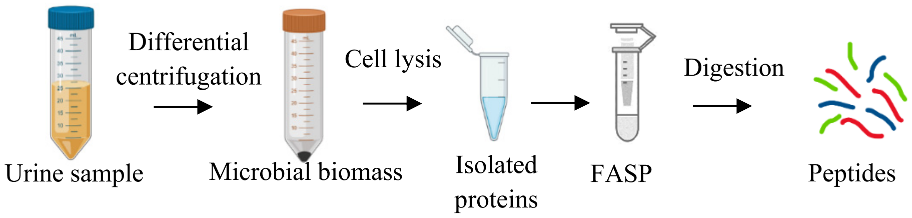

DERIVATIZATION (Step 2)

Microwave assisted derivatization of peptides

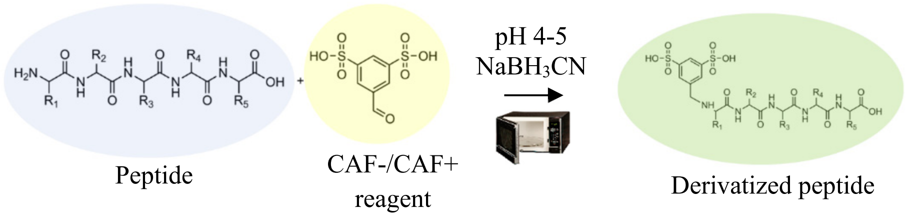

SEPARATION (Step 3)

2-D µSPE (SAX/SCX-36 fractions) automated AssayMap fractionation

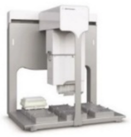

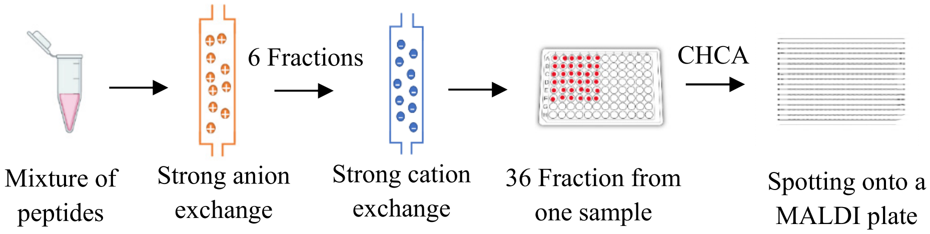

ANALYSIS (Step 4)

MALDI-TOF/TOF analysis MS negative (36 spots) followed by MS/MS negative/positive (720) selected precursor masses in each mode)

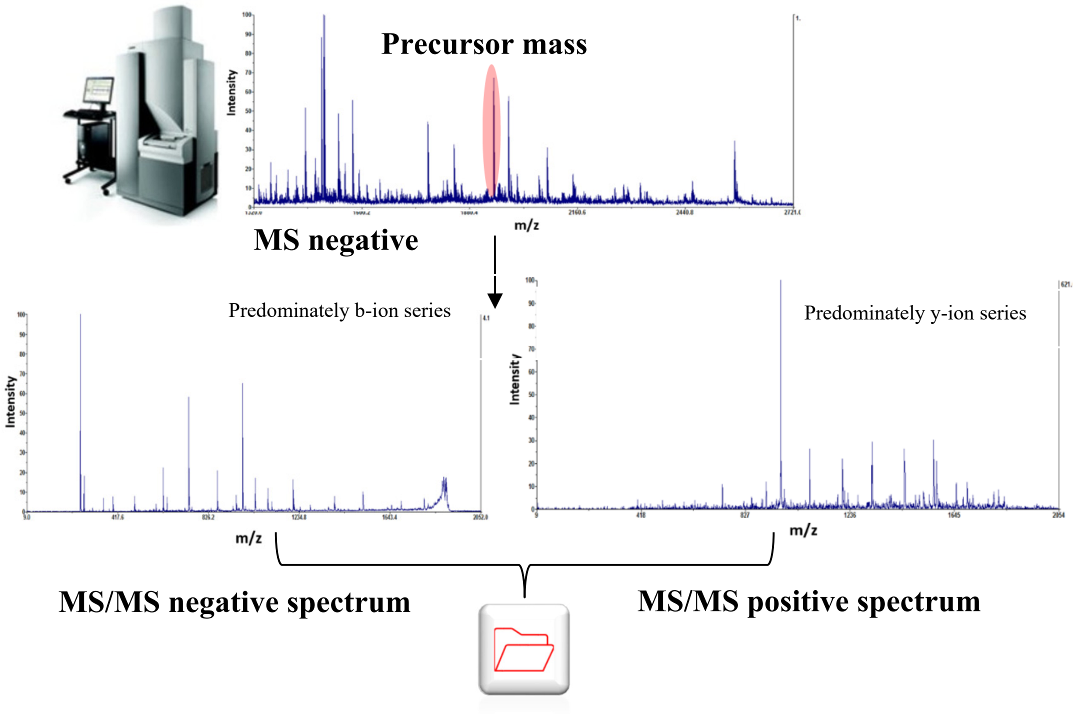

MS negative, MS/MS negative and MS/MS positive merged into one mgf file

IDENTIFICATION (Step 5)

Identification of microorganism by MALDI-MS/MS & *de novo* BLASTp using Protein Acrobat

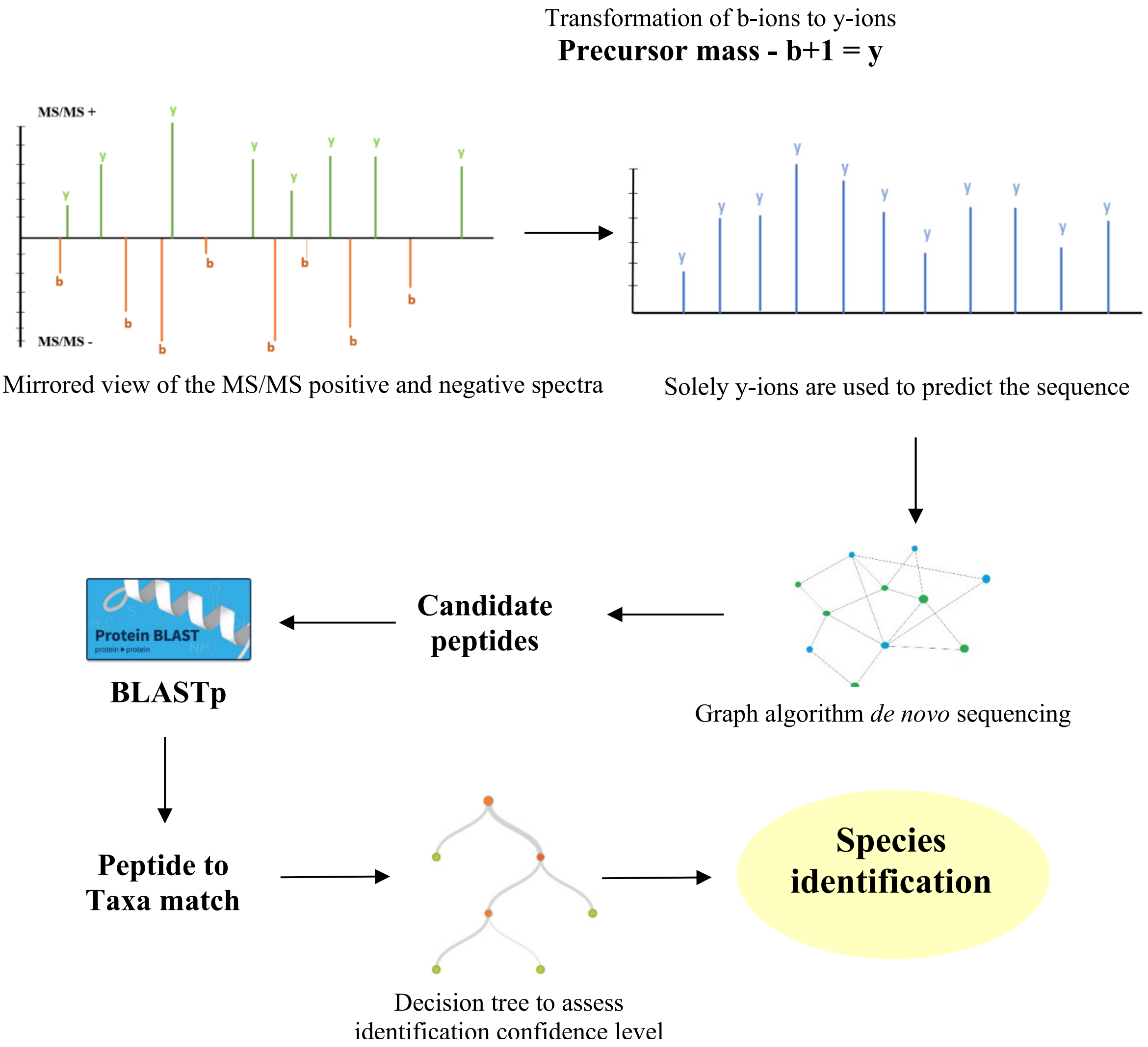

Figure S1. Workflow of five-step MALDI-MS/MS identification of urine microorganisms.

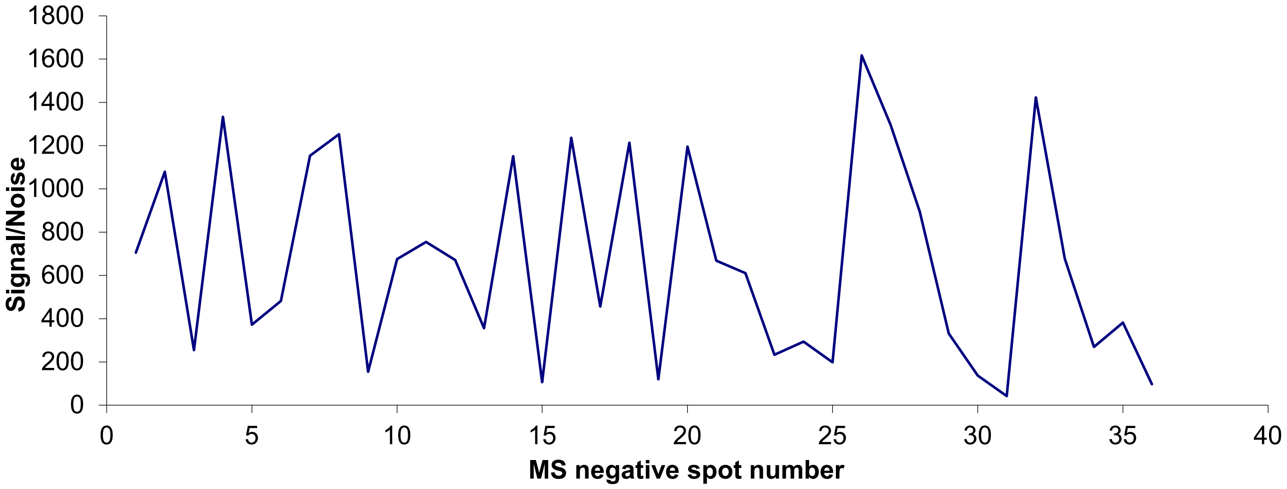

Figure S2. Mass spectra negative ion mode total signal-to-noise intensity per spot *m/z* 1000-3500.

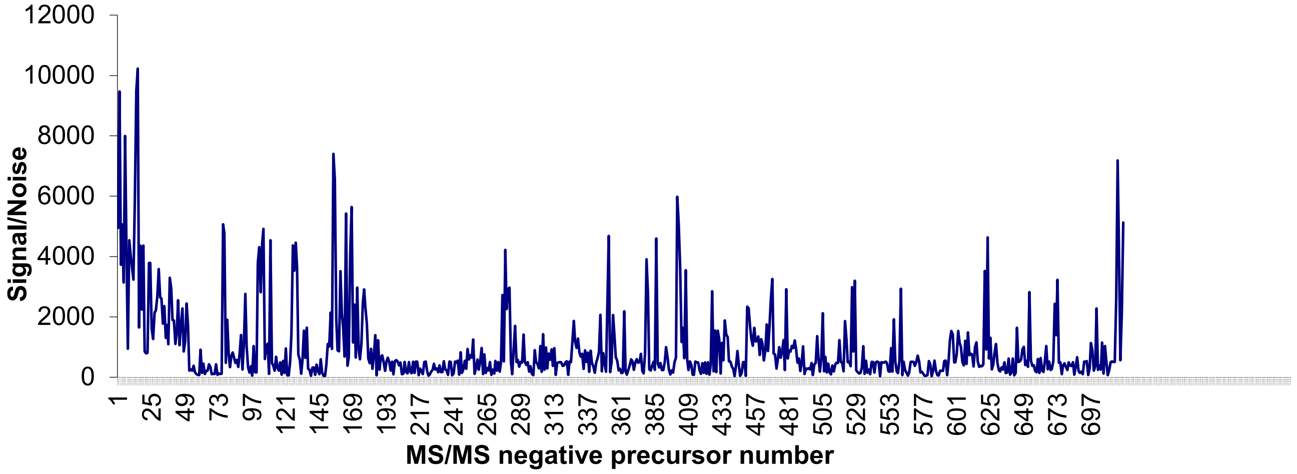

Figure S3. Tandem mass spectra negative ion mode total MS/MS signal-to-noise intensity per selected precursor ion spectrum.

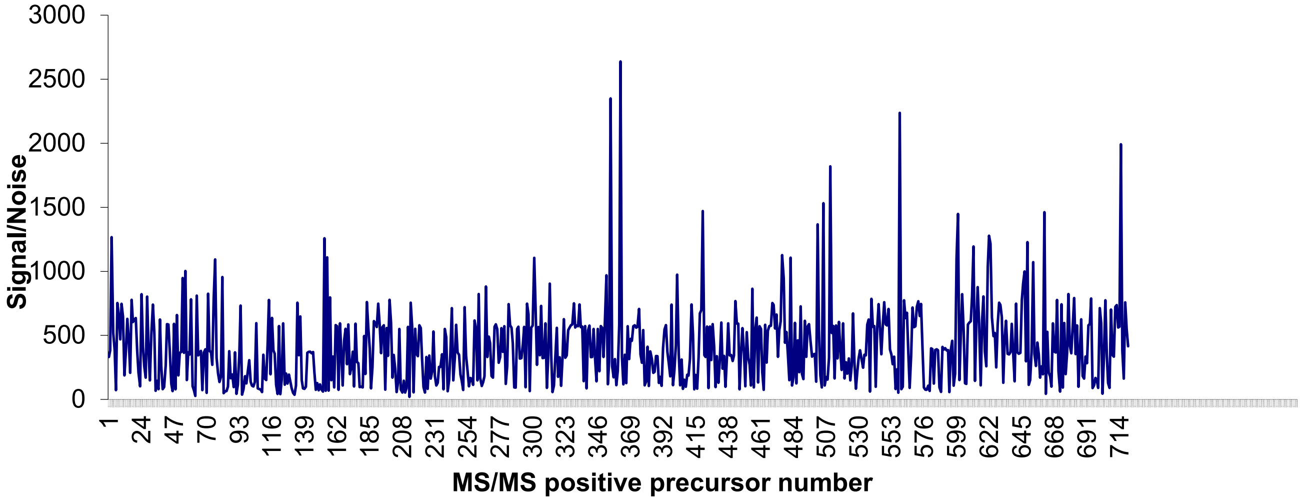

Figure S4. Tandem mass spectra positive ion mode total MS/MS signal-to-noise intensity per selected precursor ion spectrum.

Table S1: 48 common uropathogenic species adapted from Pinault et al. 2019 that were used to filter the NCBI*nr* database to enable the BLASTp search.

Number	name	preferred name	taxid
1	*Proteus mirabilis*		584
2	*Enterococcus faecalis*		1351
3	*Klebsiella pneumoniae*		573
4	*Enterobacter cloacae*		550
5	*Pseudomonas aeruginosa*		287
6	*Staphylococcus aureus*		1280
7	*Citrobacter koseri*		545
8	*Streptococcus agalactiae*		1311
9	*Staphylococcus saprophyticus*		29385
10	*Enterobacter aerogenes*	*Klebsiella aerogenes*	548
11	*Klebsiella oxytoca*		571
12	*Staphylococcus epidermidis*		1282
13	*Enterococcus faecium*		1352
14	*Morganella morganii*		582
15	*Citrobacter freundii*		546
16	*Serratia marcescens*		615
17	*Gardnerella vaginalis*		2702
18	*Proteus vulgaris*		585
19	*Stenotrophomonas maltophilia*		40324
20	*Streptococcus oralis*		1303
21	*Hafnia alvei*		569
22	*Providencia stuartii*		588
23	*Acinetobacter pittii*		48296
24	*Staphylococcus haemolyticus*		1283
25	*Pseudomonas putida*		303
26	*Staphylococcus hominis*		1290
27	*Enterobacter kobei*		208224
28	*Raoultella ornithinolytica*		54291
29	*Citrobacter amalonaticus*		35703
30	*Aerococcus urinae*		1376
31	*Acinetobacter baumannii*		470
32	*Enterococcus gallinarum*		1353
33	*Staphylococcus capitis*		29388
34	*Enterobacter asburiae*		61645
35	*Streptococcus anginosus*		1328
36	*Streptococcus mitis*		28037
37	*Streptococcus pneumoniae*		1313

**38**	*Acinetobacter nosocomialis*	106654
**39**	*Providencia rettgeri*	587
**40**	*Corynebacterium amycolatum*	43765
**41**	*Corynebacterium striatum*	43770
**42**	*Corynebacterium tuberculostearicum*	38304
**43**	*Pseudomonas plecoglossicida*	70775
**44**	*Enterobacter ludwigii*	299767
**45**	*Citrobacter braakii*	57706
**46**	*Pseudomonas mosselii*	78327
**47**	*Escherichia coli*	562
**48**	*Candida spp.*	1535326

Table S2: Buffers used for two-dimensional peptide fractionation by AssayMAP Bravo liquid handling platform. QMA= Quaternary methyl amin, ACN=Acetonitrile.

**First dimension: Strong anion exchange**	
**Stationary phase**	QMA
**Priming buffer**	400 mM NH_4HCOOH, 1% NH_4OH / 25% ACN
**Equilibration buffer**	1% NH_4OH
**Elution buffer 1**	80 mM NH_4CH_3COOH / 25% CH_3OH
**Elution buffer 2**	150 mM NH_4CH_3COOH / 25% CH_3OH
**Elution buffer 3**	200 mM NH_4CH_3COOH / 25% CH_3OH
**Elution buffer 4**	300 mM NH_4CH_3COOH / 25% CH_3OH
**Elution buffer 5**	500 mM NH_4CH_3COOH / 25% CH_3OH
**Elution buffer 6**	40 Mm NH_4HCOOH pH 3.5 / 25% CH_3OH
**Second dimension: Strong Cation Exchange**	
**Stationary phase**	SCX
**Priming buffer**	400 mM NH_4HCOOH, 1% HCOOH/ 25% ACN
**Equilibration buffer**	1% HCOOH / 25% ACN

**Elution buffer 1**	40 mM NH_4HCOOH, pH 3.5 / 25% ACN
**Elution buffer 2**	40 mM NH_4HCOOH, pH 4.0 / 25% ACN
**Elution buffer 3**	40 mM NH_4 CH_3COOH, pH 4.5 / 25% ACN
**Elution buffer 4**	40 mM NH_4 CH_3COOH, pH 5.0 / 25% ACN
**Elution buffer 5**	40 mM NH_4 CH_3COOH, pH 6.0 / 25% ACN
**Elution buffer 6**	100 mM NH_4OH, pH 9.5 / 25% ACN
